# Terahertz refractive index-based morphological dilation for breast carcinoma delineation

**DOI:** 10.1038/s41598-021-85853-8

**Published:** 2021-03-19

**Authors:** Quentin Cassar, Samuel Caravera, Gaëtan MacGrogan, Thomas Bücher, Philipp Hillger, Ullrich Pfeiffer, Thomas Zimmer, Jean-Paul Guillet, Patrick Mounaix

**Affiliations:** 1 Integration from Material to Systems Laboratory, University of Bordeaux, 33405 Talence, France; 2 Institute for High-Frequency and Communication Technology, University of Wuppertal, 42119 Wuppertal, Germany; 3 Department of Pathology, Bergonié Institute, 33076 Bordeaux, France

**Keywords:** Biophysics, Cancer, Medical research, Engineering, Optics and photonics

## Abstract

This paper reports investigations led on the combination of the refractive index and morphological dilation to enhance performances towards breast tumour margin delineation during conserving surgeries. The refractive index map of invasive ductal and lobular carcinomas were constructed from an inverse electromagnetic problem. Morphological dilation combined with refractive index thresholding was conducted to classify the tissue regions as malignant or benign. A histology routine was conducted to evaluate the performances of various dilation geometries associated with different thresholds. It was found that the combination of a wide structuring element and high refractive index was improving the correctness of tissue classification in comparison to other configurations or without dilation. The method reports a sensitivity of around 80% and a specificity of 82% for the best case. These results indicate that combining the fundamental optical properties of tissues denoted by their refractive index with morphological dilation may open routes to define supporting procedures during breast-conserving surgeries.

## Introduction

Terahertz imaging and spectroscopy have rapidly spread to different application areas thanks to the continued development of efficient emitters and detectors between 0.1 and 7-THz^1^. The biomedical field is one domain of study that could benefit from terahertz wave properties^2,3^. Radiations at terahertz frequencies have been shown to be non-ionizing and non-hazardous for biological tissues at the power commonly employed to inspect the super-cellular level^4^. Besides, terahertz radiations are notably sensitive to the presence of polar molecules such as the most abundant component of the body: water^5^. Hence, different medical topics have been assessed with terahertz imaging and spectroscopy to look for alternative and complementary methods to the existing ones. These investigations cover a broad range of possible surgical and clinical applications^6–8^. Among them, cancer diagnosis remains the most widely investigated topic throughout the literature, covering blood^9,10^, brain^11–13^, colorectal^14,15^, gastric^16,17^, liver^6^, lung^18^, oral^19^, skin^20,21^ and breast cancer^22–26^.

Investigations, conducted on breast cancer, mainly aim to develop supporting procedures for breast-conservative surgeries through breast tumour margin delineation. The success of breast-conserving surgeries is dictated by the accuracy of delineating the concentric margins of excised breast volumes. Although there is no clear description of what ideal margins are, it is recommended that no cancer cells remain adjacent to any inked edge/surface of the specimen^27^. Conserving surgeries are usually followed by postoperative radiation management to eradicate microscopic remains of disease^28^. Margin cleanliness is assessed via biopsy examinations during which excised volumes are subsequently fixed into formalin solution, embedded into paraffin, sliced in micrometric sections and immersed into different alcohol and biological stain baths. Usually, hematoxylin and eosin stains are used. The reason for that is that hematoxylin stains cell nuclei blue and eosin stains both the cytoplasm and the extracellular matrix pink. The stain draws the global layout of a tissue structure so that a pathologist judges the cleanliness of the margin^29^. Overall, two extreme cases of margin delineation can be observed: (1) positive margins—malignant cells are located at the edge of the excised volume; (2) negative margins—an absence of tumor cells at the edge or the distance of abnormal cells from the edge is at least more than 1-mm. Following histopathologic inspection, up to 20% of excised breast samples are reported to exhibit positive margins^30^. Reasons behind tumor edge delineation failure are often presence of in situ carcinoma at close proximity to the surgical margin, discontinuous tumor spread from the original surgery site, or inappropriate presurgical tumor localization and inappropriate excision during surgery^31^. A positive margin inevitably leads to a second surgery to favor low recurrence risk and to attain more widely clear surgical margins. In return, a second surgery concomitantly increases the morbidity rate.

So far, different research teams worldwide have reported the ability of terahertz imaging and spectroscopy to discriminate between healthy and malignant breast tissues. These studies were primarily conducted on formalin-fixed and paraffin-embedded breast tissue^32,33^. Such investigations opened the route for clinical studies on freshly excised breast volumes^18,22,24^. The capabilities of terahertz radiation demarcation between normal and abnormal tissue regions were originally attributed to free-water content. Indeed, free-water molecules have been proven to present a specific permittivity step around 900-GHz^5^. Moreover cancer tissues are known to exhibit a greater free-water content than normal tissues^34^. However, further studies have suggested that the origin of contrast could not be solely attributed to water. That is because specific dielectric features exhibited by breast tissues, in the low terahertz frequency band, were not observed in water dielectric profile^35^. Hence, it has been suggested that, specific functional groups play a potential role^22^. Globally, the refractive index of breast cancer tissues has been shown to be higher than the one observed for normal tissues over the terahertz band. On the contrary, the related absorption coefficient was reported as unsatisfactory parameter for demarcation^35,36^. Additionally, the contrast level between healthy and malignant tissues depends on cancer cell density. In fact, while the resolution of any light-based imager remains dictated by the diffraction limit, two objects separated by a distance less than the wavelength cannot be distinguished. For instance, the spatial resolution of a far-field imaging system operating at 1-THz will be limited to 0.3-mm. Hence, the respective response to the external terahertz radiation stimuli of two biological entities, separated by a distance smaller than 0.3-mm, will have to be averaged. Considering the typical diameter of the eukaryotic cell is at the order of tens of microns, it can be concluded that, such a terahertz imager cannot manage to resolve entities at the cellular level. It has, however, been demonstrated that the use of computational imaging system operating in a total internal reflection geometry could resolve features with a sub-wavelength lateral resolution^37^. While it can be expected that high densities of cancer cells will lead to a well-defined demarcation, the dielectric response of isolated abnormal groups may be blurred by the healthy surrounding and ultimately leading to recognition analysis failure. Although the diffraction limit of resolution may complicate recognition in areas sparsely populated by cancer cells^38^, it also raises delicate questions on the exact frontier between two well localized normal and abnormal regions. Indeed, rather than depicting a sharp contrast between areas, the obtained cliché may inevitably exhibit a smooth gradient from one to another area which is a result of class-overlapping. That is particularly limiting when it comes to providing a pixel-by-pixel diagnosis based on the information collected.

The present work proposes a new approach for the clinical classification of breast tissue pixels that overcomes the limitations aforementioned. The method is based on the extraction of the terahertz refractive index map of freshly excised samples followed by morphological dilation. A high value of the refractive index has been reported as a reliable measure of the presence of cancer within a tissue^22,24^. Morphological dilation is a part of set-theory^39^ and is commonly employed to images having characteristics of ambiguity and vagueness^40^. It consists of expanding a given shape contained in the input image. In biology, morphological processing was notably employed for counting blood cells during blood smear test^41^, to isolate female gametocyte^42^ or for skin cancer segmentation^43^.

Operating dilation from regions exhibiting a higher refractive index should allow bypassing class-overlapping limitations. Such a process is referred to as terahertz refractive index-based morphological dilation and operates as follows: (1) the refractive index map of a freshly excised breast tissue is extracted through a specific objective function minimization; (2) a refractive index threshold is defined such that pixels exhibiting a refractive index higher than the threshold are classified as malignant while others are classified as benign; (3) morphological dilation is used to spread the malignant zones to the neighborhood.

To conduct these investigations, different freshly excised breast tissues have been scanned in reflection geometry by means of a terahertz spectrometer. The refractive index maps have been extracted. Different refractive index thresholds and dilation shapes have been tested. The related pixel classifications have been compared to those provided by a pathologist. Finally, the sensitivity and specificity of each combination of threshold—dilation shape have been derived.

The paper is organized as follows: “Experimental framework” describes the experimental framework to acquire raw terahertz images of the freshly excised breast tissues. “Refractive index map” describes the mathematical background to extract the refractive index map. “Morphological dilation” defines the morphological dilation and the respective dilation shapes employed in the study. “Image registration” describes the registration of obtained images with respect to the pathological cliché. “Diagnosis compliance” details the evaluation of compliance between the classifications provided respectively by the pathologist and the reported strategy. “Results” presents the results for different samples. Finally, “Conclusions” presents the conclusions.

## Experimental framework

The experimental protocol was assessed and approved by the ethics committee of the Bergonié Institute. Human tissue analysis have been conducted in view of the fundamental ethical principles as stipulated in the Helsinki declaration and its later revisions. Written informed consent from each patient undergoing breast surgery was collected, stipulating their agreement regarding the use of their tissues for research purposes.

### Breast tissue samples

Following surgery, breast excisions were cut into slices of a few millimeters and kept into physiological serum before measurement to ensure the moisture content and delay the necrosis. A maximum of one hour elapsed between the end of surgery and the terahertz acquisition starting time. Once measurement was complete, excised tissue samples were placed in formalin-buffered solution. This process enabled the further histology routine to compare the diagnoses provided by the reported method and the pathologist. Biological samples analyzed using the method about to be reported were obtained from three different patients. One sample was excised from each of these patients.

### Measurement setup

Time-domain terahertz pulsed images were acquired with a TPS3000 spectrometer (TeraView Ltd, Cambridge, UK) operating in reflection geometry. In such systems, terahertz pulses are generated from the activation of a GaAs photoswitch. A photoswitch consists of a discontinuous metallic antenna patterned onto a photoconductive layer. Ultra-fast near-infrared pulses with an energy greater than the semiconductor band gap are focused onto the gap between the two electrodes forming the photoswitch. The incident pump laser thus propagates within the photoconductive layer and generates electron–hole pairs due to absorption. Those photocarriers are then accelerated within the electric field of the biased antenna. The acceleration of these charges produces a transient current that drives the metallic antenna and is eventually emitted as a broadband terahertz pulse. The bandwidth directly depends on the lifetime of the carriers before recombination. The carrier lifetime in the GaAs crystal is in the subpicosecond scale, hence enabling pulses with a bandwidth ranging from 200-GHz to 2-THz.

**Figure 1:**
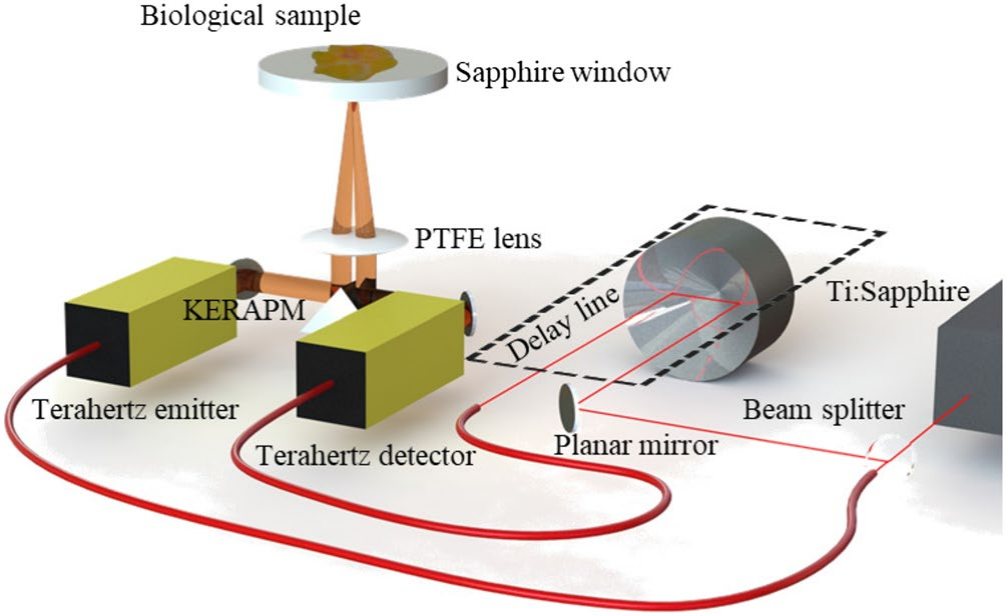
Schematic of the acquisition system. Drawn on SolidWorks 2020 SP3, www.solidworks.com.

The schematic of the experimental set-up is given in Fig. 1. The route of the terahertz pulses is governed by two planar mirrors and a knife-edge right-angle prism mirror (KERAPM). The terahertz pulses are focused on the tissue sample supported by a 2-mm thick non-birefractive C-cut sapphire substrate (see Supplementary Information, Supplementary Fig. 1) via a polytetrafluoroethylene (PTFE) lens. The maximum incident angle of the terahertz pulses is $10^{\circ }$. Both the reflections at the air-sapphire and sapphire-tissue interfaces are then focused onto a photoconductive antenna detector. The detector is sourced from the same ultra-fast near-infrared pulses used for terahertz wave generation with a beam splitter. The pulses are, however, delayed in time with a mechanical delay line. The periodic variation of the delay line length allows a time gated detection of terahertz pulses reflected by the object. In order to reduce the natural absorption of terahertz pulses by water vapor molecules, the terahertz route is confined within nitrogen chamber.

## Refractive index map

To extract the refractive index from a raw frequency image, a reference electric field has to be recorded. The reference electric field $E^r(\omega )$ refers to the electric field generated by the acquisition system. The reference measurement records the electric field of the reflection from a metal plate that is located where the sapphire substrate sample holder is aimed to be positioned for tissue imaging. From the reference electric field $E^r(\omega )$, the experimental transfer function $T^s(\omega )$, which is a measure of the disturbance experienced by the incident field as a result of the interaction with the sample, can be calculated:

1 $$\begin{aligned} T^s(\omega ) = \frac{E^s(\omega )}{E^r(\omega )}, \end{aligned}$$

with $E^s(\omega )$ the sample frequency-dependent electric field. The shape of transfer function $T^s(\omega )$ is a function of the refractive index $n(\omega )$ and the extinction coefficient $\kappa (\omega )$ of the sample under inspection. $E^s(\omega )$ depends on the Fresnel’s coefficients in transmission $T(\omega )$ and in reflection $R(\omega )$, and on propagation coefficients $P(\omega ,d)$:

2 $$\begin{aligned} E^s(\omega ) \propto T_{air-sapphire}(\omega ) \times R_{sapphire-tissue}(\omega ) \times T_{sapphire-air}(\omega ) \times P^2_{sapphire}(\omega ,d), \end{aligned}$$

with *d* being the thickness of the sapphire substrate. The Fresnel’s coefficients $T(\omega )$ and $R(\omega )$, as well as propagation terms $P(\omega ,d)$ relate to the refractive index $n(\omega )$ and the extinction coefficient $\kappa (\omega )$ through:

3a $$\begin{aligned} T_{a-b}(\omega )&= \frac{2{\hat{n}}_a}{\hat{n}_a + {\hat{n}}_b}, \end{aligned}$$

3b $$\begin{aligned} R_{a-b}(\omega )&= \frac{{\hat{n}}_a - {\hat{n}}_b}{{\hat{n}}_a + {\hat{n}}_b}, \end{aligned}$$

3c $$\begin{aligned} P_{a}(\omega ,d)&= e^{-j\frac{\omega d}{c}{\hat{n}}_a}, \end{aligned}$$

where *a* and *b* are the indices of the respective medium, $\hat{n}$ is the complex refractive index defined as $\hat{n}= n(\omega ) - j\kappa (\omega )$ and *c* is the light velocity in vacuum. Although the extinction coefficient $\kappa (\omega )$ is involved in the calculation of the transfer function $T^s(\omega )$, no significant differences have been reported in the literature between normal and abnormal tissue extinction^22,35^. Hence, solely the refractive index is further considered as a possible intrinsic parameter for demarcation.

### Map extraction

The extraction of the complex refractive index $\hat{n}(\omega )$ at each pixel from the experimental transfer function $T^s(\omega )$ can be performed by solving an inverse electromagnetic problem. Inverse electromagnetic problems usually minimize a specific convex objective function. This function denotes the discrepancies between the experimental waveform $E^s(\omega )$ and the waveforms $E^c_x(\omega )$ successively computed from a set of candidate parameters, where the *x*-index refers to the $x^{th}$-candidate tested. The candidate waveforms $E^c_x(\omega )$ are computed as stipulated in^44^. The corresponding transfer functions $T^c_x(\omega )$ are calculated in the same way as described by (1). The measures of discrepancies $\delta M_x(\omega )$ between the experimental transfer function $T^s(\omega )$ and the computed transfer functions $T^c_x(\omega )$ are defined as:

4 $$\begin{aligned} \delta M_x(\omega ) = ln\left( \frac{|T^s(\omega )|}{|T^c_x(\omega )|}\right) . \end{aligned}$$

The natural logarithmic ratio is favored here instead of standard difference as it is more penalizing. Finally, the objective function $\chi (\omega )$ to be minimized is defined as:

5 $$\begin{aligned} \chi (\omega ) = \delta M(\omega ) \times \delta M(\omega ). \end{aligned}$$

The minimization of the transfer function is subject to the following set of candidate parameters:

6 $$\begin{aligned} \begin{aligned} {\underset{n(\omega ), \kappa (\omega )}{min}} \chi (\omega ), \text {subject to } \left\{ \begin{array}{ll} n \in [1.5 ; 3], with \;\; \Delta n = 1.10^{-2}\\ \kappa \in [0 ; 1], with \;\; \Delta \kappa = 1.10^{-3}\\ \end{array} \right\} \end{aligned} \end{aligned}$$

It was stated before that the sample is maintained by the sapphire substrate. Instead of extracting the properties of the sapphire substrate for each pixel, the properties were extracted upstream, in absence of a sample, and following the same minimization process. The sapphire properties are provided in Supplementary Information, see Supplementary Fig. 2. Finally, applying the above process to each electric field stored in each pixel of the sample image allows to construct the refractive index map.

**Figure 2:**
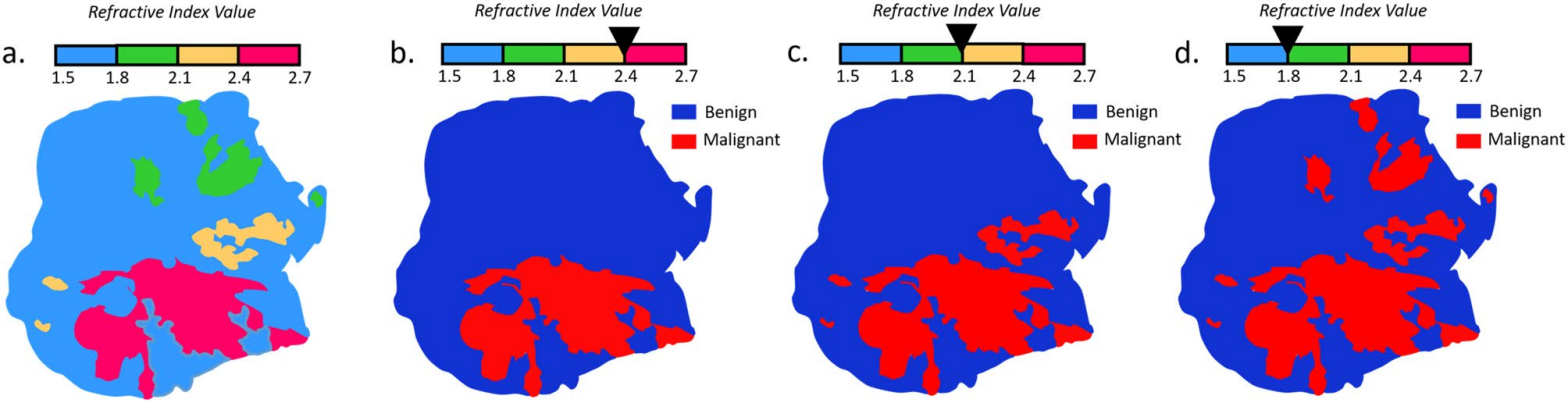
Thresholding principle applied to the refractive index map. (**a**) Schematic refractive index map; (**b**) binary refractive index map with a threshold set at 2.4; (**c**) binary refractive index map with a threshold set at 2.1; (**d**) binary refractive index map with a threshold set at 1.8.

Once the refractive index map is obtained, it is converted to a binary map that shows areas that are considered malignant or benign. To do so, a threshold among the refractive index vector has to be set. Depending on the defined value for the threshold, one may progressively increase or decrease the extent of areas classified as malignant, since pixels with a refractive index higher than the threshold are classified as cancerous. A schematic of the process is given in Fig. 2.

### Operating frequency

Although the refractive index is often referred to as optical constant, its profile varies as a function of the frequency. Previous studies have reported the terahertz frequency dependent refractive index values of abnormal and normal breast tissues^36^. Overall, the global difference between these values was shown to be the highest between 300- and 700-GHz, roughly. Hence, rather than investigating the entire band, the classification was operated at 550-GHz, as a good trade-off between signal-to-noise ratio (SNR) and higher frequency spatial resolution^45^. However, naively classifying pixels via the refractive index exhibited at 550-GHz may hardly be relevant. In particular, the refractive index extracted at the edges of malignant regions with low density may present values close to the ones of healthy tissues. Therefore, morphological dilation is introduced to overcome this limitation.

## Morphological dilation

**Figure 3:**
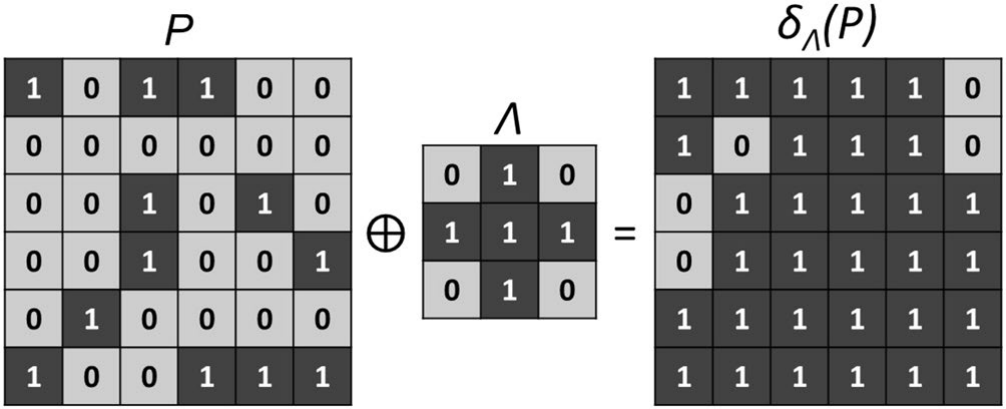
Morphological dilation operated with a cross structuring element $\Lambda$ on the matrix *P*.

Prior to dilation, the refractive index map is converted to a binary image as it was described in the previous section. The dilation can therefore be referred to as binary dilation. The dilation consists of a shift-invariant addition, denoted “$\oplus$”, within the meaning of Minkowski^46^. Mathematically, let’s define *P* as an ensemble that contains the pixels (*x*, *y*) of the tissue imaged. The binary dilation $\partial _\Lambda (P)$ of *P* by a shape $\Lambda \in \mathbb {Z}^2$ - also referred to as a structuring element, is given by:

7 $$\begin{aligned} \partial _\Lambda (P) = P \oplus \Lambda = \left\{ x + \lambda , y + \lambda | \lambda \in \Lambda \right\} , \end{aligned}$$

where $\lambda \in \Lambda$ produces the translation from *P* to $\partial _\Lambda (P)$. Supposing the matrix *P* and the structuring element $\Lambda$ as represented in Fig. 3, the matrix $\partial _\Lambda (P)$ is obtained by superimposing the center of $\Lambda$ aligned with each pixel in *P* that has a value of 1.

**Figure 4:**
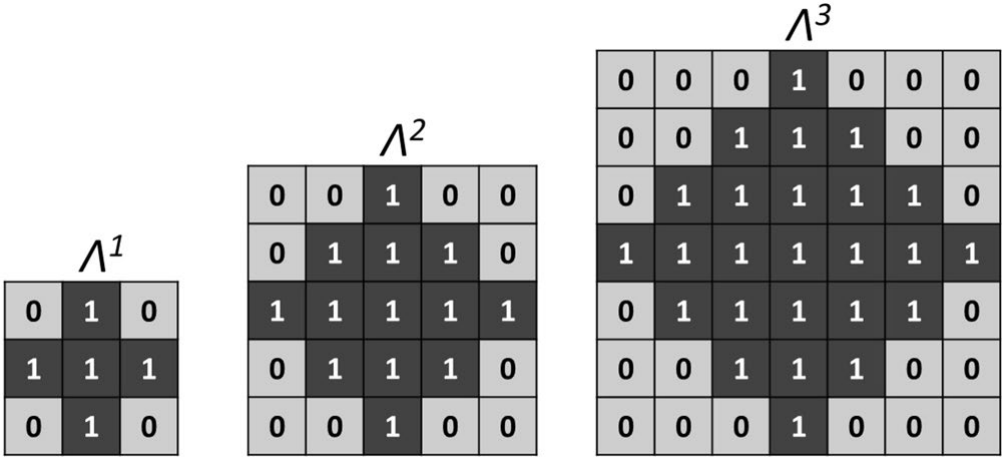
Geometry of the three different classifiers $\Lambda ^1$, $\Lambda ^2$, $\Lambda ^3$.

In the present work, three different structuring elements have been considered to dilate the binary refractive index map. They are referred to as $\Lambda ^1$, $\Lambda ^2$ and $\Lambda ^3$ classifiers. Their spatial properties are exposed in Fig. 4. These specific geometries allow the classifiers to act in the close vicinity of a starting pixel and with the same impact in all directions.

Therefore, depending on the classifier considered, a pixel may be attributed to the malignant group if at least one of the component $\lambda$ of the structuring element $\Lambda ^n$—where $n \in \mathbb {N}^{*}$—reports a pixel whose refractive index is higher than the defined refractive index threshold. Alternatively, the structuring elements can be seen as the area of influence of a cancerous pixel. Consequently pixels with a refractive index lower than the threshold but situated in such an area of influence, are turned into malignant pixels. It is however important to note that the process is constrained to a unique dilation and therefore, newly classified malignant pixels cannot, in turn, exercise a zone of influence.

**Figure 5:**
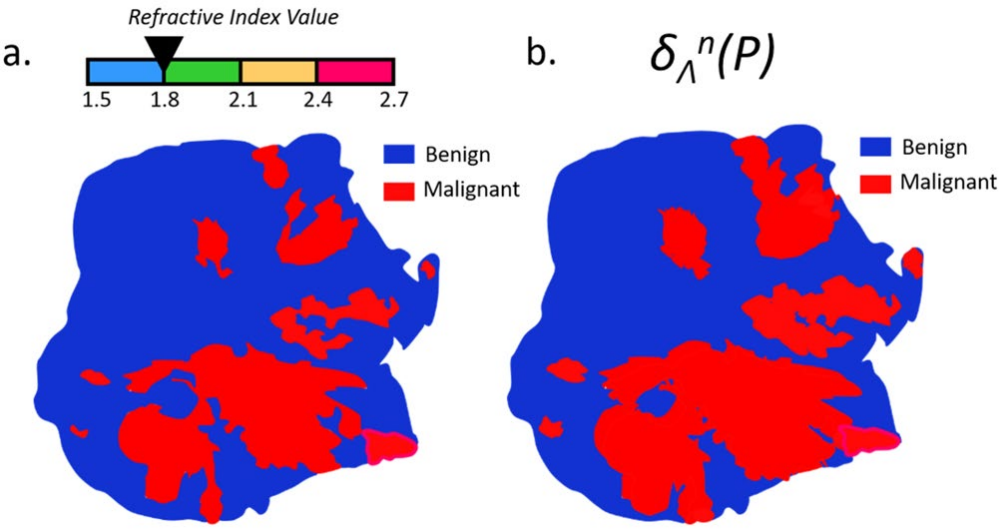
Schematic of a morphological dilation applied to a binary refractive index map over a tissue sample. (**a**) Binary refractive index map with a threshold set at 1.8; (**b**) morphological dilation applied to the binary refractive index map with an arbitrary classifier $\Lambda ^n$.

In order to carry out the dilation and the registration steps that follow, it is essential to preserve the morphology of the imaged sample. To do this, the dilation procedure must be carried out with respect to the initial contour of the sample generated from a standard contouring algorithm, thus preventing the appearance of cancerous pixels outside the original surface of the sample. A schematic of the dilation process operated on a binary refractive index map is given in Fig. 5.

## Image registration

The classification images provided by the reported method and the ones given by the pathologist do not share the same coordinate system. Image registration is the process of migrating different images into one common coordinate system^47^. Therefore, image registration is necessary to enable the comparison between the data sets. Effectively, the spatial resolution of the optical microscope used to acquire pathology clichés is far greater than the one of the employed terahertz imager. Additionally, the orientation of the tissue sample in the terahertz image and in the clinical image are expected to be different, as they are not acquired with the same angle. A simple pixel-by-pixel comparison is therefore not possible as it stands. Prior to comparison, images have to be resized and reorientated. The registration process is feature-based and solely involves image contours to avoid unintentional human bias. The different steps that are followed to register the images with respect to each other are hereafter described.

### Contouring

Contour lines, also called isolines, can be calculated by interpolating the value of the scalar field found at each pointel of each pixel. An infinite number of isolines can however be delineated. The choice of the contour to define the spatial extent of the sample in the image remains therefore subjective. For each sample, the isoline that suited the visualized tissue area best was determined by carefully comparing the terahertz image and the different contour levels.

### Resizing

As the resolution of the images is different, it is necessary to resize the histology pictures. To do so, a bicubic interpolation is operated onto pathology images. Contrary to the previous interpolation, where it is based on the four nearest pixels, bicubic interpolation takes into account a neighborhood of sixteen pixels. Therefore, bicubic interpolation provides a smoother histology slide than simple bilinear interpolation.

### Reorientation

**Figure 6:**
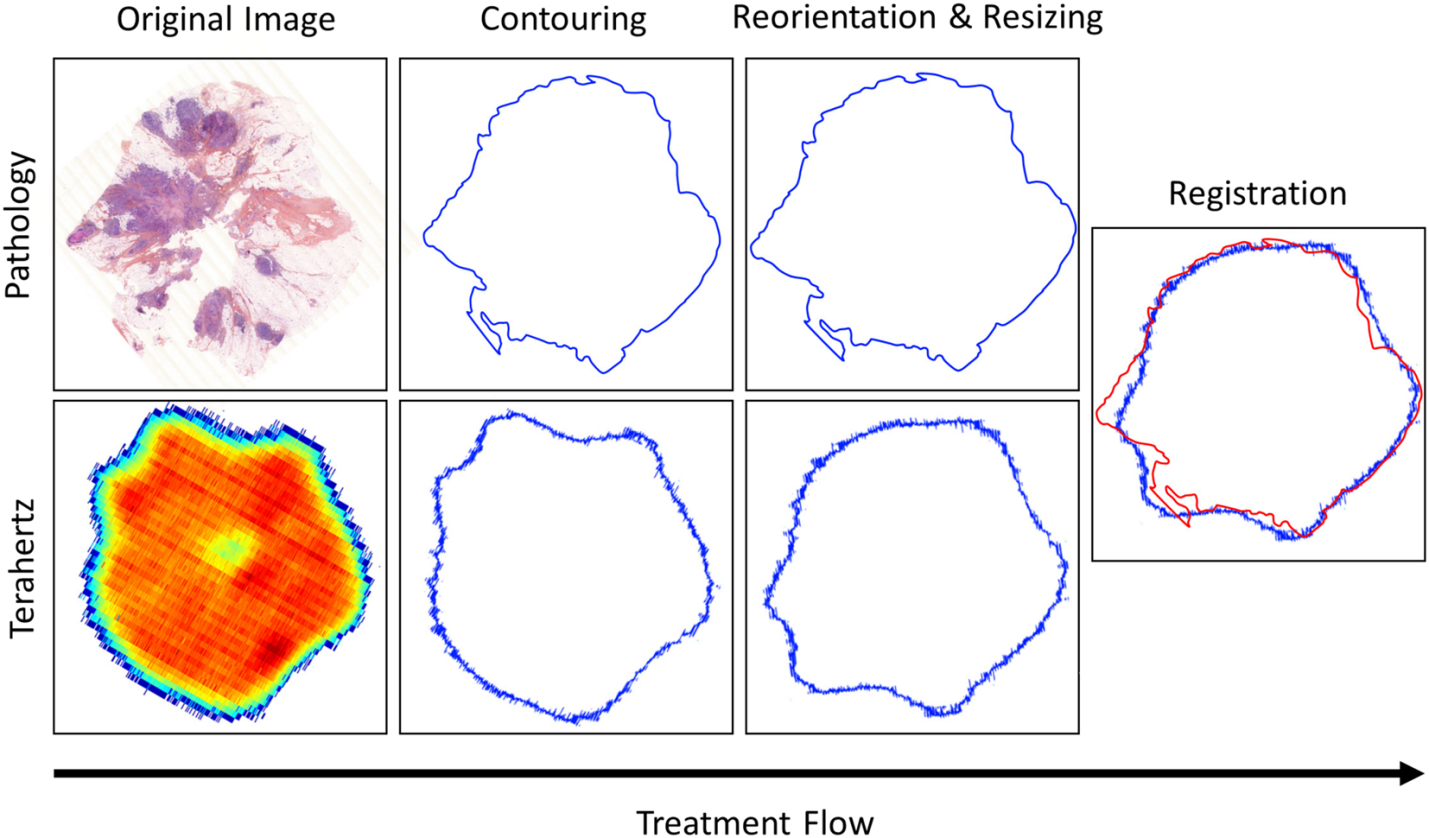
Flow chart of the registration procedure for predicted diagnosis evaluation.

First, the contour of the terahertz image is manually and progressively twisted to bring it closer to the twist angle of the pathology contour. Once the orientations approximately match, the pathology contour is iteratively rotated to establish the correlation between the two contour matrices at each step. Basically, it consists in determining the Pearson’s correlation coefficients^48^. The rotation angle providing the highest positive correlation is selected and the terahertz image is correspondingly rotated. The flow chart of these three pre-treatments, namely contouring, resizing and reorientation for image registration is provided in Fig. 6.

### Image discrepancies issues

Although one can resize and reorientate the two images with respect to each other, the pathology cliché and the terahertz image may not perfectly depict the same information. First, while terahertz imaging is performed directly on freshly excised tissues, the pathology diagnosis is established after the histology routine. Moreover, to obtain the pathology image, the excised tissue is first fixed in neutral buffered formalin, then dehydrated in subsequent alcohol baths with increasing concentrations, then cleared in a solvent before being infiltrated and finally embedded in paraffin wax. At this stage, the processed tissue is encased in a paraffin block that can be sliced in sections of a few microns thickness to be deposited on glass slides. These tissue sections are deparaffinized, rehydrated and subsequently stained with hematoxylin and eosin dyes. Finally, they are dehydrated in alcohol and cleared in a solvent before being mounted with a coverslip. The embedding, the sectioning and the desiccation alter the global structure of the tissues. These alterations are collectively referred as artefacts^49^. Artefacts include loss of tissue area and details, folds and wrinkles or cracks and holes. These alterations may result in misinterpretation as they are modifying the morphological structure of tissues. Alternatively, these artefacts may drastically limit the evaluation of the terahertz classification compliance (see Supplementary Information Supplementary Fig. 3, for an example based on one of the tissue reported by the present work). However, histological slides remain the only available reference picture that allows one to examine the performances of classifier under-test. Overall, there are two ways to deal with such issues: (1) correcting the histology slides at risk of adding artificial information; (2) comparing directly the terahertz image with the raw pathology image at risk of underestimating the efficiency of the method. The first way would require to morph the pathology image to correspond to the terahertz picture. Some procedures to do so were reported in the literature^50^. However, these methods are cumbersome and the evaluation of the histological cliché reconstruction is often complicated since no perfect reference pathology image exists. As terahertz imaging remains a new technology for breast carcinoma delineation, the second approach was favored—at risk of underestimating the efficiency of the classifiers.

## Diagnosis compliance

Following the histology routine, pathology images are colored in different shades of blue and pink. The pathologist draws the contour of malignant areas based on his/her expertise. From the interpretation of the pathologist, the images were binarized and each pixel was classified either as benign or as malignant^51^.

Once both diagnosis images exhibit binary information, have the same size and orientation, the compliance between them can be evaluated. In case of discrepancies, the pathologist classification prevails over terahertz delineation. The present section describes how the ability of classifiers was evaluated with respect to the pathologist one.

### Performance of the classification test

As each diagnosis presents a binary information, four different cases can be distinguished:

- True negative: both methods classify a pixel as benign;
- True positive: both methods classify a pixel as malignant;
- False positive: the terahertz method stands for a malignant pixel while histology stipulates a benign pixel;
- False negative: the terahertz method stands for a benign pixel while histology stipulates a malignant pixel.

Hence, for each refractive index threshold associated with a specific classifier, one can fill the corresponding confusion matrices that highlight the classification procedure performances. In such error matrices, the rows represent the instances in the terahertz class, here the predicted class, while columns represent the actual diagnosis provided by histology examination^52^.

From these matrices, the effectiveness of the classification method is assessed by creating the receiver operatic characteristic (ROC) curve^53^ for each classifier. The ROC curve represents the ability of the classifier to provide the correct diagnosis as the refractive index threshold varies. The ROC curve is obtained by plotting the true positive rate (TPR) as a function of the false positive rate (FPR). The TPR is defined as the number of true positives divided by all pixels classified by the pathologist as positives: true positives and false negatives. The FPR is defined as the number of false negatives divided by all pixels classified by the pathologist as negatives: false positives and true negatives. It can also be thought as a plot of the sensitivity—that is equivalent to the TPR defined in Eq. (8), against the probability of false-alarm—that can be calculated as (1—specificity) and defined in Eq. (9)^54^. These measures of performances are favored as they are not sensitive to changes in data distributions, compared to accuracy and to error rate. Hence, both metrics can be used with imbalanced data^55^.

8 $$\begin{aligned}&\text {True Positive Rate} = \text {Sensitivity} = \frac{\text {True Positives}}{\text {True Positives} + \text {False Negatives}}, \end{aligned}$$

9 $$\begin{aligned}&\text {False Positive Rate} = 1 - \text {Specificity} = \frac{\text {False Positives}}{\text {False Positives} + \text {True Negatives}}. \end{aligned}$$

To complement these measures, the area under each ROC curve (AUC) is calculated as it relies on the performance of score classifiers for all possible classification thresholds^56^.

Finally, the best discrimination thresholds are selected as the ones that provide the highest sensitivity while preserving the healthy tissue area from false diagnosis, i.e. specificity. It is noted that the aforementioned classification procedure is studied for the specific case of breast conserving surgery. Hence, it is essential to preserve the healthy area while removing the malignant zones. Ultimately, the best classifiers are selected as the ones that provide the highest measure of $TPR - FPR$, since higher values of this function indicates more accurate results.

## Results

In this section, the classifiers are employed to evaluate their effectiveness on three freshly excised breast tissues. Two of these samples were diagnosed as invasive ductal carcinoma (IDC) and one was identified as an invasive lobular carcinoma (ILC). These samples are referred to as test sample TS#1, TS#2 and TS#3.

### TS#1

**Figure 7:**
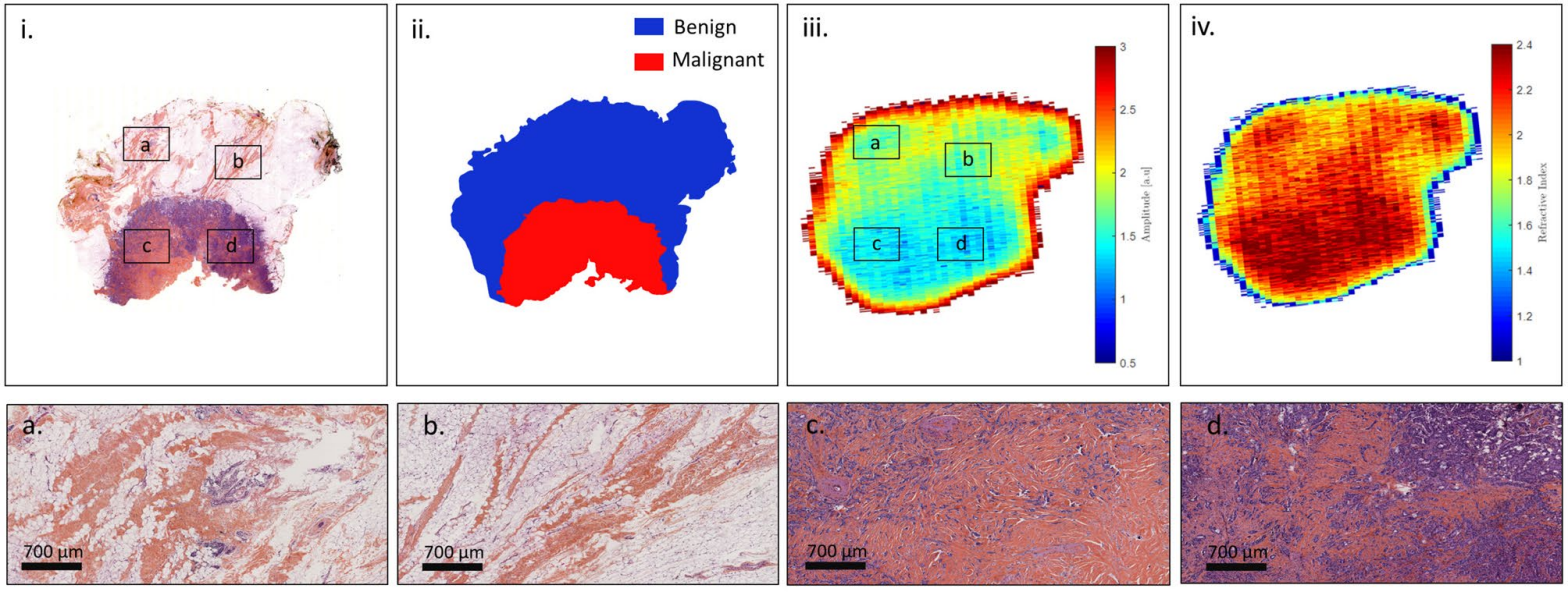
Sample TS#1. (i) Pathology image and correlated view of the respective zones (**a**,**b**,**c**,**d**); (ii) pathology mask; (iii) raw terahertz image at 550-GHz; (iv) refractive index map at 550-GHz.

TS#1 is an invasive ductal carcinoma. The pathology image with some enlightened pathology areas, the pathology mask, the raw terahertz image obtained at 550-GHz and the correlated refractive index map are presented in Fig. 7.

**Figure 8:**
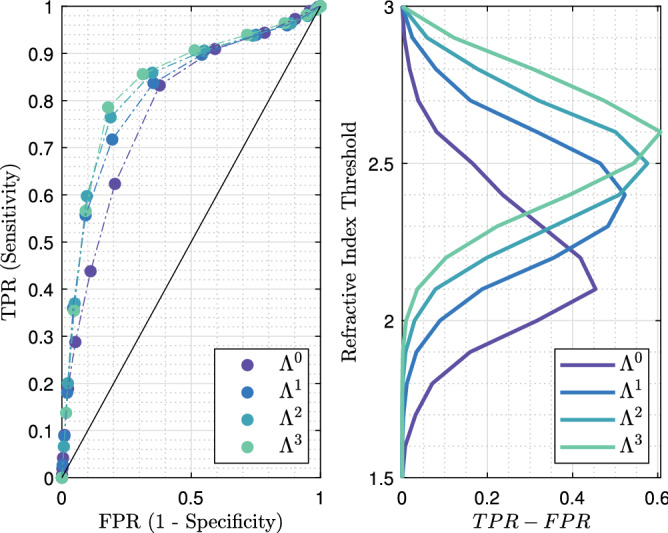
Left: receiver operating characteristic for the different classification methods, at 550-GHz applied to TS#1. The black line stands for $TPR = FPR$. Right: refractive index threshold as a function of the $TPR - FPR$ measure for the different classifiers.

It can be observed that the raw terahertz image as well as the refractive index map exhibits specific features that correspond to the pathology image. Regions depicted in Fig. 7a,b. correspond to fibrous tissues that are included in an adipose matrix. Such regions are therefore expected to globally give rise to a lower refractive index than the one classified as malignant as depicted in Fig. 7c,d. Although such a refractive index seems overall lower than the refractive index of the tumour, it remains relatively close to it. Therefore, classifying only on the basis of the refractive index would certainly prove to be inefficient. The sensitivity and the specificity of each structuring element classifier for varying refractive index threshold were calculated for TS#1. The corresponding ROC curves and $TPR-FPR$ functions are given in Fig. 8.

Each $\Lambda ^n$-dependent ROC curve is located to the left of the $TPR = FPR$ line in Fig. 8, proving that the fraction of true positives is greater than the proportion of false positives. It is clear that the use of the refractive index alone as a classifier ($\Lambda ^0$) is shown to be less efficient than associating the refractive index with a classifier. Such a statement is not surprising as the classification does not consider the neighborhood. While on ROC graphics, depicted in Fig. 8, it does not seem that obvious which classifier among $\Lambda ^1$, $\Lambda ^2$ and $\Lambda ^3$ performs well, the $TPR-FPR$ visualization indicates that the structuring element $\Lambda ^3$ in association with a high refractive index threshold by about 2.6 is the most efficient rule of classification. The association provides a classification with a sensitivity by around 80% and a specificity of 82%. What is more, the wider the structuring element, the higher the refractive index has to be set for good performances. Effectively, starting with a high refractive index makes it possible to identify, in a first instance, tissue areas densely populated with cancer cells, while a broad structuring element makes it possible to efficiently spread the identification over a wide zone.

The corresponding AUC for each ROC curve, the $TPR - FPR$ value, the sensitivity and the specificity for the first two best refractive index thresholds are given in Table 1 (see Supplementary Information, Supplementary Table 1. for the complete list of performances). While $\Lambda ^1$ and $\Lambda ^2$ are less efficient than $\Lambda ^3$ for both sensitivity and specificity, the $\Lambda ^0$ classifier provides a slightly greater sensitivity for a threshold of 2.1, by about 83%. However, the gain of 4% in sensitivity with respect to $\Lambda ^3$ costs concomitantly 20% in method specificity. Reasonably, this gain is not worth it, considering such a drastic decrease in classification specificity. Alternatively, if one wants to increase the sensitivity while maintaining specificity at a reasonable level, second best thresholds may offer a promising substitute. On using the second best threshold provided by $\Lambda ^3$ of 2.5, an increase of 7% in sensitivity conjointly leads to a decrease by about 12% in specificity. By doing so, one reaches a sensitivity of 86%.

**Table 1:** Statistical measure of the performance of the classifiers and AUC.

Classifier	$\Lambda ^0$		$\Lambda ^1$		$\Lambda ^2$		$\Lambda ^3$	
AUC	0.7804		0.8149		0.8285		0.8360	
RI-threshold	2.1	2.2	2.4	2.3	2.5	2.4	2.6	2.5
TPR–FPR	0.4540	0.4181	0.5227	0.4829	0.5759	0.5093	0.6068	0.5433
Sensitivity%	83	62	72	84	76	86	**79**	86
Specificity%	62	79	81	65	81	65	**82**	69

The sensitivity and specificity obtained for the best performing classifier-refractive index threshold association is given in bold.

**Figure 9:**
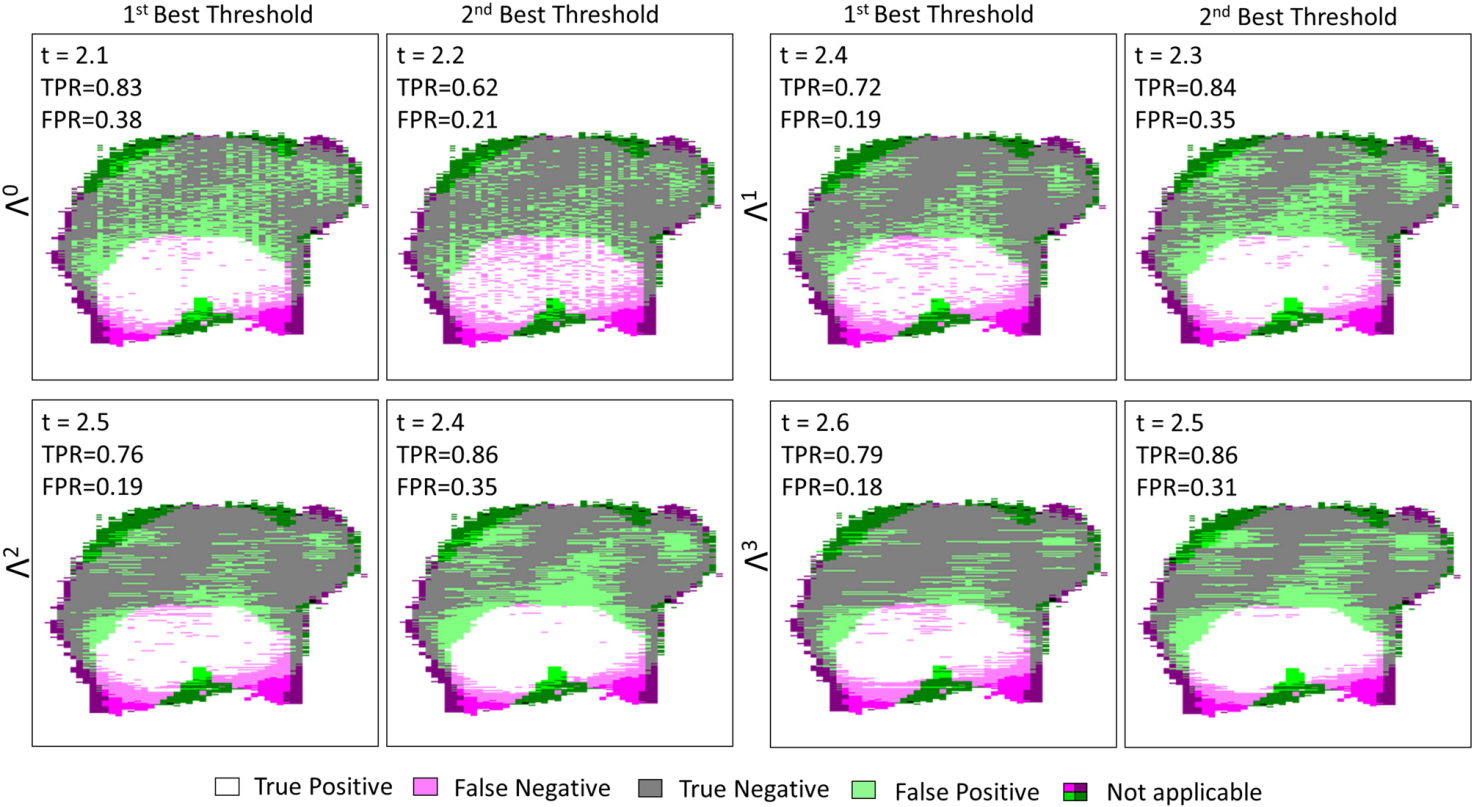
TS#1 tissue sample classification maps at 550-GHz for $\Lambda ^0$, $\Lambda ^1$, $\Lambda ^2$, $\Lambda ^3$ and their respective first two best thresholds. “Not applicable” refers to regions where the binary pathology classification and the binary terahertz classification image do not match spatially. The values listed in each box are respectively standing for the refractive index threshold, the true positive rate and the false positive rate.

The superimposition of the classification images from the reported method and the clinical one, corresponding to the performances listed in Table 1 are given in Fig. 9.

### TS#2

**Figure 10:**
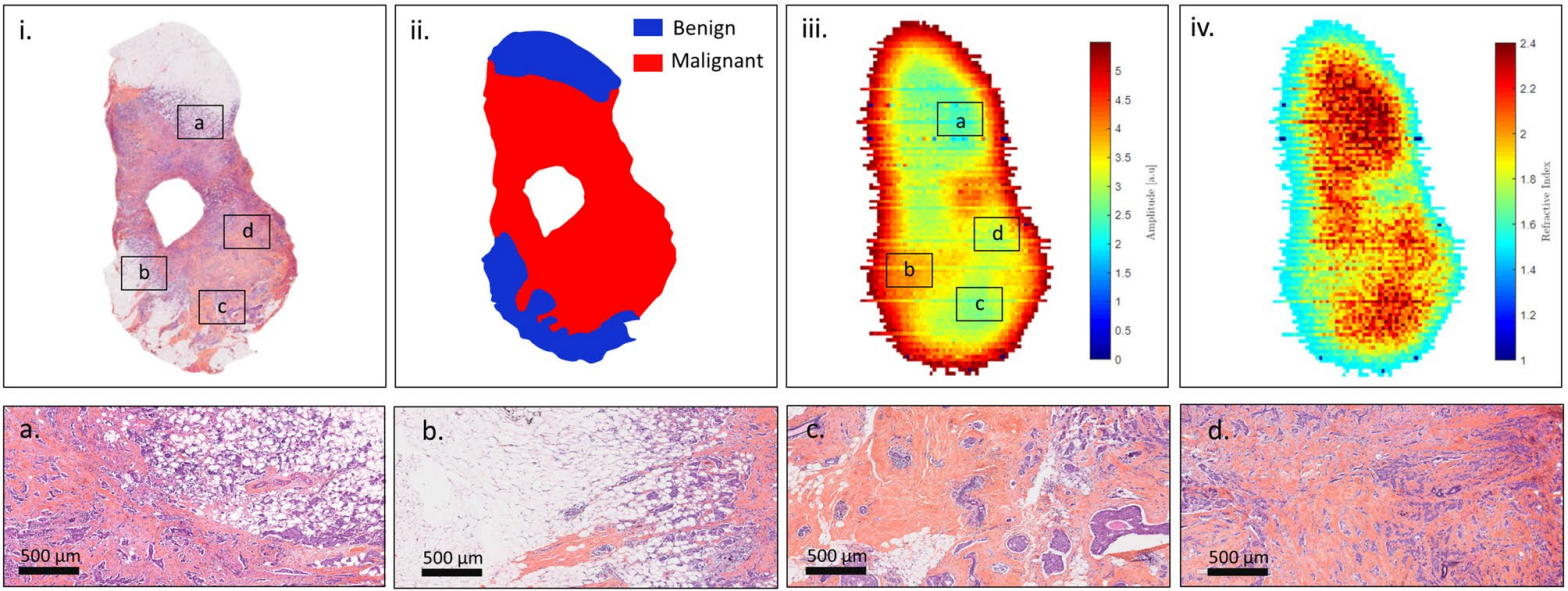
Sample TS#2. (i) Pathology image and correlated view of the respective zones (**a**,**b**,**c**,**d**); (ii) pathology mask; (iii) raw terahertz image at 550-GHz; (iv) refractive index map at 550-GHz.

TS#2 sample is an invasive ductal carcinoma from which a 67 years old woman was suffering. The initial tumor site was found to be roughly 100 mm$^2$. On Fig. 10, the pathology image with some enlightened pathology areas, the pathology mask, the terahertz image at 550-GHz, and the refractive index map are shown. The pathology image as well as the pathology mask exhibit the presence of a hole, where no tissue is found. The lack of tissue in the middle of the section is not natural and enlightens the issues, that have been previously reported towards pathology images. Hence, this specific region is not considered for performance evaluation.

**Figure 11:**
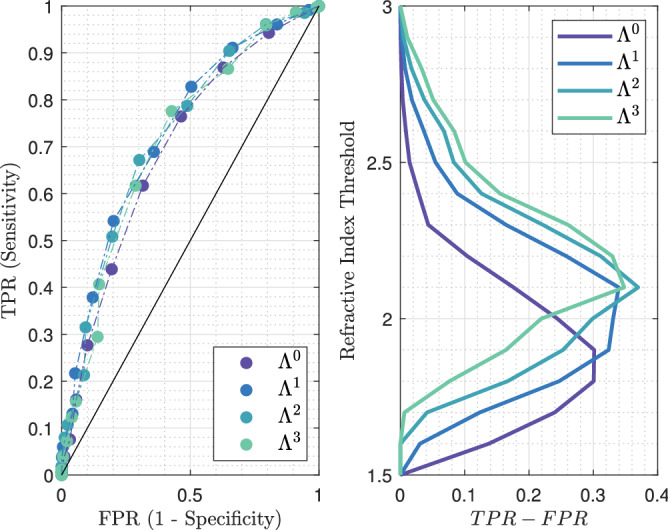
Left: receiver operating characteristic for the different classification methods, at 550-GHz applied to TS#2. The black line stands for $TPR = FPR$. Right: refractive index threshold as a function of the $TPR - FPR$ measure for the different classifiers.

The ROC curves as well as the $TPR - FPR$ function for different classifiers with various thresholds are given in Fig. 11. Similarly to the foregoing, all ROC curves are located to the left of the $TPR = FPR$ line, hence proving that the fraction of true positives remains greater than that of false positives.

The most effective classifiers towards conserving classification are $\Lambda ^2$ and $\Lambda ^3$, both for a threshold set at 2.1. While the combination of such a threshold with $\Lambda ^2$ provides a sensitivity of 67% and a specificity of 70%, the same threshold operating with $\Lambda ^3$ gives rise to a sensitivity by about 78% and a specificity of 57%. Hence, tuning the structuring element geometry would offer an interesting trade-off between specificity and sensitivity. The respective performances of each classifier applied to TS#2 are listed in Table 2 (see Supplementary Information, Supplementary Table 1 for the complete list of performances).

**Table 2:** Statistical measure of the performance of the classifiers and AUC.

Classifier	$\Lambda ^0$		$\Lambda ^1$		$\Lambda ^2$		$\Lambda ^3$	
AUC	0.6976		0.7307		0.7264		0.7127	
RI-threshold	1.9	1.8	2.1	2.0	2.1	2.2	2.1	2.2
TPR–FPR	0.3013	0.3006	0.3395	0.3307	0.3697	0.3113	0.3480	0.3295
Sensitivity%	62	76	54	69	**67**	51	78	62
Specificity%	68	54	80	64	**70**	80	57	71

The sensitivity and specificity obtained for the best performing classifier-refractive index threshold association is given in bold.

**Figure 12:**
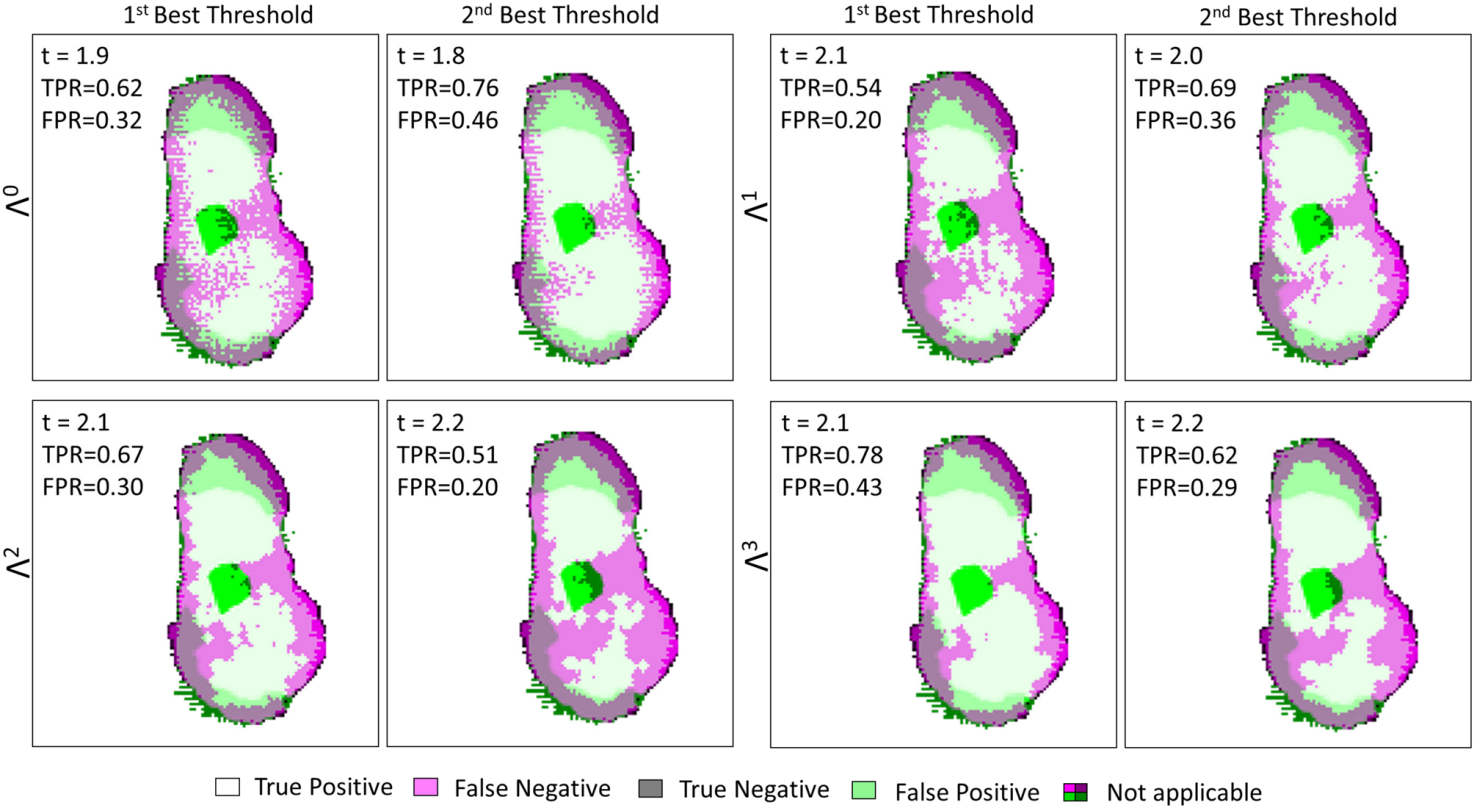
TS#2 tissue sample classification maps at 550-GHz for $\Lambda ^0$, $\Lambda ^1$, $\Lambda ^2$, $\Lambda ^3$ and their respective first two best thresholds. “Not applicable” refers to regions where the binary pathology classification and the binary terahertz classification image do not match spatially. The values listed in each box are respectively standing for the refractive index threshold, the true positive rate and the false positive rate.

The classification maps involving each classifier and their respective best performing thresholds are exposed in Fig. 12. These images show the improvement in classification with the use of morphological dilatation. Moreover, they highlight the difficulties of good prediction at the outer margins. Low performance at the outer margins may come from the non-conformity of the information in these areas between the terahertz image and the histology picture. The most convincing hypothesis for this non-conformity is the tissue deformation imposed by the histological routine.

### TS#3

**Figure 13:**
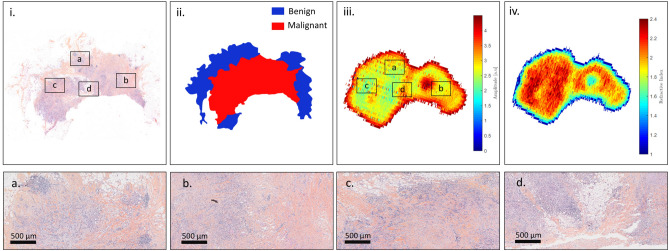
Sample TS#3. (i) Pathology image and correlated view of the respective zones (**a**,**b**,**c**,**d**); (ii) pathology mask; (iii) raw terahertz image at 550-GHz; (iv) refractive index map at 550-GHz.

In contrast to the two previous samples, the TS3 sample was taken from an 83 years old patient with an invasive lobular carcinoma. On Fig. 13, the pathology image with some enlightened pathology areas, the pathology mask, the terahertz image at 550-GHz, and the refractive index map are shown.

**Figure 14:**
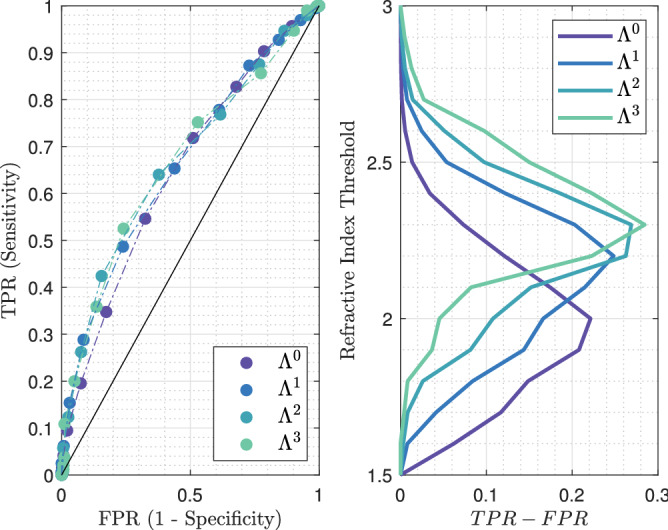
Left: receiver operating characteristic for the different classification methods, at 550-GHz applied to TS#3. The black line stands for $TPR = FPR$. Right: refractive index threshold as a function of the $TPR - FPR$ measure for the different classifiers.

The ROC curves plotted in Fig. 14 indicate a lower efficiency towards classification than the efficiencies for TS#1 and TS#2. The cause may be found in the distribution of cancer cells within the malignant zone, in comparison to previously tested samples. While for other cases the malignant zone was densely populated, cancer cells are found in small quantity and in an inhomogeneous manner over TS#3. Additionally, the histology routine may have altered the tissue morphology as stated in “Image discrepancies issues”.

The AUC values and the performances for each classifier are given in Table 3 (see Supplementary Information, Supplementary Table 1. for the complete list of performances). Despite the lower efficiency the most accurate classifying strategy remains $\Lambda ^3$ when associated with a refractive index threshold of 2.3. The $TPR - FPR$ measure is by around 0.28 with a sensitivity of 53% and a specificity of 76%. As already indicated these performances are below the ones reached for other study cases. $\Lambda ^2$ classifier offers a greater specificity of 85% but simultaneously concedes 11% upon sensitivity, thus falling below the critical threshold of half the number of malignant pixels correctly classified. The weak density of cancer cells within a lobular carcinoma slice may lead one to opt for $\Lambda ^0$ classifier operating in association with a low refractive index threshold to maximize the sensitivity, despite a concomitant loss in specificity.

**Table 3:** Statistical measure of the performance of the classifiers and AUC.

Classifier	$\Lambda ^0$		$\Lambda ^1$		$\Lambda ^2$		$\Lambda ^3$	
AUC	0.6478		0.6631		0.6695		0.6693	
RIThreshold	2.0	1.9	2.2	2.1	2.3	2.2	2.3	2.4
TPR–FPR	0.2215	0.2080	0.2484	0.2148	0.2690	0.2626	0.2845	0.2234
Sensitivity%	55	72	49	65	42	64	**53**	36
Specificity%	68	49	76	56	84	62	**76**	86

The sensitivity and specificity obtained for the best performing classifier-refractive index threshold association is given in bold.

**Figure 15:**
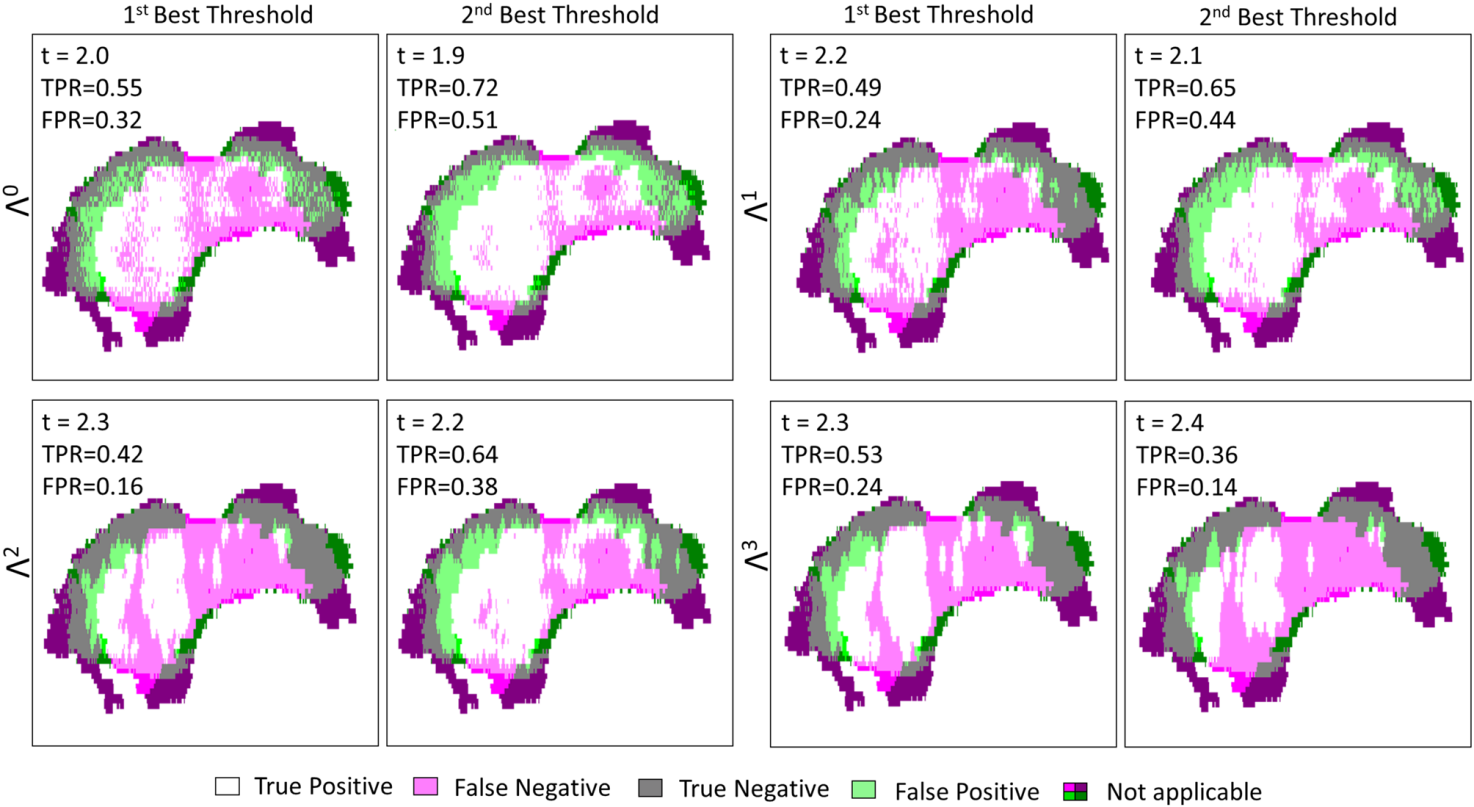
TS#3 tissue sample classification maps at 550-GHz for $\Lambda ^0$, $\Lambda ^1$, $\Lambda ^2$, $\Lambda ^3$ and their respective first two best thresholds. “Not applicable” refers to regions where the binary pathology classification and the binary terahertz classification image do not match spatially. The values listed in each box are respectively standing for the refractive index threshold, the true positive rate and the false positive rate.

The corresponding classification images for TS#3 for each classifier and the correlated best refractive index threshold are demonstrated in Fig. 15. The global classification clearly suffers from the spatial discrepancies between the fresh state tissue and the histological state. Even though such differences are expected to be the main roots behind classification accuracy weakness, the histological type of TS#3 may also trigger difficulties. It can be assumed that the classification strategy may provide better performances when applied on ductal carcinoma cases than on lobular ones. However, it is noted that the number of samples investigated does not allow to assert such a hypothesis.

## Conclusions

In this paper, a new approach to support breast carcinoma margin delineation during surgeries with terahertz radiations was proposed. The method relies on the acquisition of the excised samples by means of a terahertz time-domain imager followed by a segmentation based on the extracted refractive index map at 550-GHz and its morphological dilation. Morphological dilation was introduced to overcome the weakness of the refractive index alone as a classifier in tissue regions sparsely populated with cancer cells. Dilation was used to construct a zone of influence of pixels. Hence, tissue areas close to regions identified as malignant were succesfully classified as cancerous despite a refractive index suggesting benign zones.

The performances of the classifications were assessed for three different samples. Overall, the association of a high refractive index threshold with a wide dilation has shown to be the most appropriate combination to maintain both method sensitivity and specificity at decent levels for invasive ductal carcinoma. The best performances of the methods have been reported to stand by about 80% in sensitivity and 82% in specificity. On the contrary, the same methodology applied onto an invasive lobular carcinoma showed lower performances. Various hypothesis were drawn to determine the roots for classification failure. While lobular carcinoma are globally less populated by cancer cells than the ductal histology type, pathology image alterations may also have contribute by rendering the diagnosis evaluation tedious.

The recognition performances of malignant areas could be improved. Indeed, the terahertz classification has localized false negatives surrounded by true positives. Therefore, implementing an additional and simple processing that classifies as malignant, benign-predicted pixels that are encircled by cancerous ones would enhance the classification accuracy.

Although investigations on higher rank classifier, i.e. for $\Lambda ^n$ with $n > 3$, have not been conducted, a more efficient structuring element could be found. Nevertheless, a high rank for a structuring element is accompanied by an equally high refractive index threshold. Thus, a reasonable assumption would be that the refractive index suitable for the use of these higher-ranked classifiers lies beyond the optical properties of biological tissues.

This preliminary investigation towards terahertz refractive index-based morphological dilation may open the routes to refine strategies to improve the accuracy with which breast tumour margins are delineated. However, the field still stands in its early stages and suffers challenges due to pathology reference image alterations that complicate classification correctness assessment. Additionally, performance comparison with other classification algorithms are yet to be investigated and will be needed to pursue with the proposed methodology. Finally, and in authors’s opinion, the applicability of terahertz waves for breast carcinoma margin demarcation still requires further studies to evaluate its feasibility in the clinical environment.

## Methods

Numerical procedures were conducted with in-house software, written with the MatLab development framework. The software follows mathematical procedures described in this paper and our preceding works.

## Supplementary Information

Supplementary Information
