# Supplementary material for: Terahertz refractive index-based morphological dilation for breast carcinoma delineation
Source: Sci Rep. 2021 Mar 19;11:6457. doi: 10.1038/s41598-021-85853-8 (PMC7979939; doi:10.1038/s41598-021-85853-8)
Supplement: Supplementary file 1 — Supplementary Information [file 41598_2021_85853_MOESM1_ESM.pdf]

Terahertz Refractive Index-based Morphological Dilation for Breast Carcinoma Delineation

Quentin Cassar[1], Samuel Caravera[3], Gaëtan MacGrogan[3], Thomas Bücher[2], Philipp Hillger[2], Ullrich Pfeiffer[2], Thomas Zimmer[1], Jean-Paul Guillet[1], and Patrick Mounaix[1,*]

[1]Integration: from Material to Systems Laboratory, University of Bordeaux, Talence, 33405, France
[2]Institute for High-Frequency and Communication Technology, University of Wuppertal, Wuppertal, 42119, Germany
[3]Bergonié Institute, Department of Pathology, Bordeaux, 33076, France
[*]patrick.mounaix@u-bordeaux.fr

ABSTRACT

This paper reports investigations led on the combination of the refractive index and morphological dilation to enhance performances towards breast tumour margin delineation during conserving surgeries. The refractive index map of invasive ductal and lobular carcinomas were constructed from an inverse electromagnetic problem. Morphological dilation combined with refractive index thresholding was conducted to classify the tissue regions as malignant or benign. A histology routine was conducted to evaluate the performances of various dilation geometries associated with different thresholds. It was found that the combination of a wide structuring element and high refractive index was improving the correctness of tissue classification in comparison to other configurations or without dilation. The method reports a sensitivity of around 80% and a specificity of 82% for the best case. These results indicate that combining the fundamental optical properties of tissues denoted by their refractive index with morphological dilation may open routes to define supporting procedures during breast-conserving surgeries.

Supplementary Information

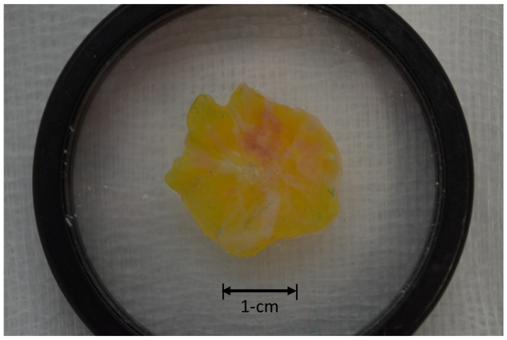

Figure 1. Photograph of a freshly excised breast tissue mounted on the sapphire window.

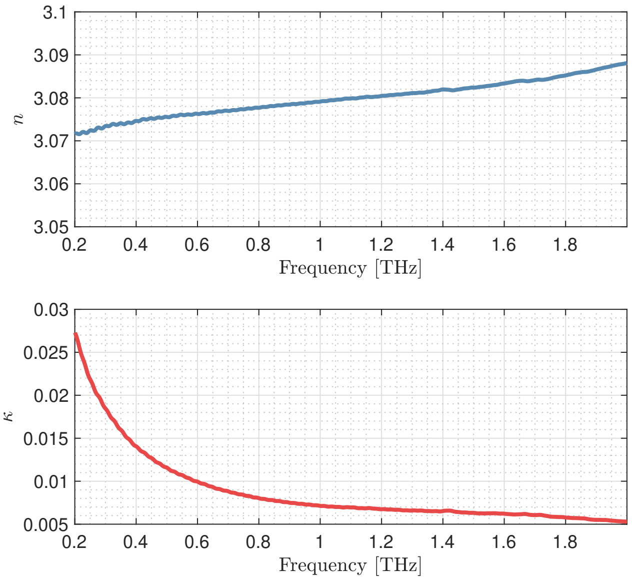

Figure 2. Frequency-depend optical properties of the 2-mm C-cut sapphire substrate used to maintain breast tissue during acquisition. Top: frequency-dependent refractive index profile of the sapphire substrate. Bottom: frequency-dependent extinction coefficient behavior of the sapphire substrate. At 550-GHz, $\hat{n} \approx 3.076 - j0.0108$.

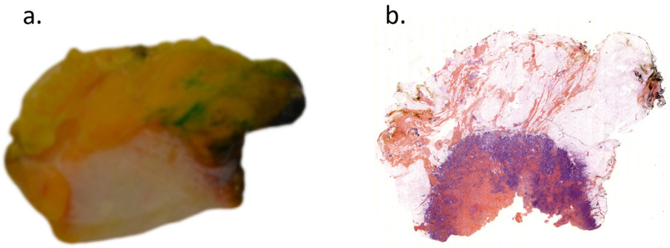

Figure 3. Morphological differences between tissue in the fresh-state and pathology image. a: Photograph of the TS#1 sample. b: Pathology image of the TS#1 sample. Clear evidence of tissue shrinkage and hardening is observed, thus complicating the registration and the evaluation of the diagnosis established by the reported classification method.

Table 1. Statistical measure of classifier performances as a function of different refractive index thresholds for TS#1, TS#2 and TS#3.

TS#1								
Classifier	Λ^0		Λ^1		Λ^2		Λ^3	
Threshold	Sens.-%	Spec.-%	Sens.-%	Spec.-%	Sens.-%	Spec.-%	Sens.-%	Spec.-%
2.9	0.6	> 99.9	2.7	99.7	6.6	99.3	13.8	98.4
2.8	1.9	99.8	8.7	99.1	20.0	97.7	35.4	95.3
2.7	4.2	99.6	18.0	98.0	37.0	95.1	56.6	90.8
2.6	9.1	99.0	36.0	95.7	59.7	90.4	78.6	82.1
2.5	18.9	97.6	55.7	90.8	76.4	81.2	85.6	68.7
2.4	28.8	94.8	71.7	80.5	85.9	65.0	90.7	48.7
2.3	43.8	88.9	83.7	64.6	90.6	44.9	94.0	28.4
2.2	62.3	79.5	89.7	45.9	93.8	26.1	96.4	13.9
2.1	83.2	62.2	93.9	24.8	96.4	11.4	98.1	5.4
2.0	91.0	40.8	96.0	12.9	97.9	5.0	99.2	1.7
1.9	94.4	21.6	98.2	5.1	99.5	1.3	99.8	0.2
1.8	97.3	9.8	99.5	1.6	> 99.9	0.2	> 99.9	< 0.1
1.7	> 99.9	4.1	> 99.9	0.4	> 99.9	< 0.1	> 99.9	< 0.1
1.6	> 99.9	0.8	> 99.9	< 0.1	> 99.9	< 0.1	> 99.9	< 0.1
TS#2								
Classifier	Λ^0		Λ^1		Λ^2		Λ^3	
Threshold	Sens.-%	Spec.-%	Sens.-%	Spec.-%	Sens.-%	Spec.-%	Sens.-%	Spec.-%
2.9	< 0.1	> 99.9	0.2	> 99.9	0.5	> 99.9	1.1	> 99.9
2.8	0.2	> 99.9	0.7	> 99.9	1.9	99.8	3.8	99.5
2.7	0.4	> 99.9	1.8	> 99.9	4.1	99.6	7.2	97.9
2.6	0.9	> 99.9	3.8	99.8	8.0	98.8	12.3	96.1
2.5	1.6	99.8	6.0	99.5	10.7	97.6	15.7	94.5
2.4	3.9	99.0	13.1	95.8	21.3	91.3	29.5	86.0
2.3	7.6	96.8	21.7	94.8	31.5	90.7	40.7	85.4
2.2	16.1	94.3	37.9	87.9	50.8	80.3	61.7	71.3
2.1	27.7	90.0	54.2	79.8	67.2	69.8	77.6	57.2
2.0	43.9	80.5	68.9	64.2	78.7	51.2	86.6	35.3
1.9	61.7	68.4	82.8	49.6	90.4	34.9	95.9	20.5
1.8	76.5	53.6	91.1	33.6	96.1	20.6	98.7	9.0
1.7	86.9	37.1	96.1	16.3	98.5	5.6	99.7	0.9
1.6	94.3	19.5	99.2	3.9	99.8	0.1	> 99.9	< 0.1
TS#3								
Classifier	Λ^0		Λ^1		Λ^2		Λ^3	
Threshold	Sens.-%	Spec.-%	Sens.-%	Spec.-%	Sens.-%	Spec.-%	Sens.-%	Spec.-%
2.9	< 0.1	> 99.9	0.2	> 99.9	0.6	99.5	1.2	99.3
2.8	< 0.1	> 99.9	0.3	> 99.9	1.0	99.5	2.0	99.3
2.7	0.2	> 99.9	0.8	> 99.9	2.0	99.4	3.8	99.0
2.6	0.5	> 99.9	2.5	> 99.9	5.7	99.4	10.9	98.9
2.5	1.5	99.8	6.2	99.2	12.3	97.4	20.0	95.0
2.4	4.1	99.3	15.4	96.9	26.2	92.4	35.9	86.5
2.3	9.5	97.9	28.8	91.5	42.4	94.5	52.5	75.9
2.2	19.5	92.6	48.7	76.2	64.0	62.2	75.2	47.1
2.1	34.7	58.7	65.3	56.2	76.8	38.4	85.6	22.6
2.0	54.6	67.5	77.8	38.8	87.5	23.2	94.7	9.8
1.9	71.8	49.0	87.3	27.1	94.7	13.5	99.0	4.7
1.8	82.7	32.1	92.7	15.7	98.0	4.6	99.9	0.9
1.7	90.3	21.4	97.0	7.2	99.7	1.1	> 99.9	0.4
1.6	95.7	10.5	99.5	1.3	99.9	0.3	> 99.9	< 0.1
